# A Pragmatic Perspective of the Antibacterial Properties of Metal-Based Nanoparticles

**DOI:** 10.3390/nano11123214

**Published:** 2021-11-26

**Authors:** Edward Sacher, Arthur Yelon

**Affiliations:** Département de Génie Physique, Polytechnique Montréal, CP 6079, Succursale Centre-Ville, Montréal, QC H3C 3A7, Canada; arthur.yelon@polymtl.ca

**Keywords:** antibacterial efficacy, corona functionalization, epitope mediation, nanoparticle alloys, surface component

## Abstract

A consideration of the antibacterial efficacy of metal-based nanoparticles, from the point of view of their physicochemical properties, suggests that such efficacy arises from the protein coronas that form around them, and that the contents of the coronas depend on the chemical groups found on the nanoparticle surfaces. We offer a new perspective and new insights, making use of our earlier observations of the physicochemical properties of nanoparticle surfaces, to propose that the nanoparticle serves as a mediator for the formation and activation of the protein corona, which attacks the bacterium. That is, the nanoparticle enhances the body’s natural defenses, using proteins present in body fluids.

## 1. Introduction

In today’s society, there is a pervasive view that, no matter what ails one, there is a pill for that. This, and the undue consumption of antibiotics in animal husbandry and fish farming, together with the extensive use of pesticides and herbicides in agriculture, have led to the overuse and, ultimately, abuse of medications. Such abuse has given rise to bacterial mutations that are impervious to the antibiotics once used to overcome them; as an example, although methicillin is used to treat *Staphylococcus aureus*, the medical community also recognizes the existence of *methicillin-resistant Staphylococcus aureus*. The crisis this situation has created has provoked a search for new methods to combat diseases; among them is the renewed consideration of metals, particularly in the form of nanoparticles.

There is a long history of the use of metals as antibiotics. Copper, for example, has been used as such for at least 4000 years [1] and silver, even longer [2]. Indeed, a perusal of the literature [3] indicates that many metal-based nanoparticles have antibacterial properties. The mechanisms thought to underlie such properties have been reviewed [3], including those for the Ag, Cu systems already mentioned, and their mixtures and alloys [4]. The reader is directed to references [3] (196 references) and [4] (286 references) for detailed discussions of the various toxicity mechanisms and pertinent references. A characteristic of all the studies referred to in these reviews is the assumption of the highest purity for all the nanoparticles considered, from core to surface.

Basing ourselves on several decades of work that we have carried out on the physicochemical characterizations of First, Second and Third Transition Series metal nanoparticles and their surfaces, we know that this assumption is untrue: all the nanoparticle surfaces are heavily oxidized, and also contain adventitious carbon. Thus, it is the chemical structure of the real surface that should concern us, not the zerovalent metal. It is our purpose here to use our earlier results to discuss the real nanoparticle surface, the groups that are present there, and how they may convey antibacterial properties. We do not pretend to offer a final view of how bacteria are attacked by metal nanoparticles: there are too many avenues yet unexplored for us to do that. We offer, instead, suggestions as to the avenues that need to be considered.

## 2. Nanoparticle Surface Oxidation

Chemical thermodynamics indicates that reactions occur when the change in Gibbs free energy is negative. At surfaces, the Gibbs free energy is replaced [5,6] by the Helmholtz free energy, which is directly related to the surface tension. That is, the reactions occurring at surfaces lower the surface tension. Thus, metals, having surface tensions ranging to 3000 mJ m^−2^, will react with the ambient atmosphere to reduce their surface tensions. This is, in fact, why metals corrode and why adventitious carbon (oxidized carbon-containing materials deposited from the vacuum background) deposits onto samples under vacuum.

We have studied the surfaces of nanoparticles of First (e.g., Cu [7]), Second (e.g., Ag [8]) and Third (e.g., Pt [9]) Transition Series metals. Each was deposited onto freshly cleaved highly oriented pyrolytic graphite, under a vacuum of ~10^−10^ torr, in a baked-out X-ray photoelectron spectrometer (XPS), and analyzed without exposure to atmosphere. In all cases, despite being deposited under high vacuum, the nanoparticle surfaces were both highly oxidized and covered with adventitious carbon. Indeed, in the case of commercial Ti [10], the oxide layer, on receipt, was so thick that zerovalent Ti could not be seen by XPS, indicating that the oxide layer thickness was greater than 4.5 nm, the probe depth at ~795 eV, the kinetic energy of the Ti 2p_3/2_ peak.

It is thus clear that, given the opportunity, the metal at the nanoparticle surface will react with ambient gases, so as to reduce its surface energy. This being so, there seems little likelihood that bare metal surfaces exist for any length of time, and certainly not in practical applications.

## 3. The Antibacterial Properties of Metal Oxides

Given the fact that the surfaces of metal nanoparticles are oxidized, it behooves us to consider whether such surfaces have antibacterial properties. The antibacterial efficacies of many metal oxides, such as CuO [11] and Ag_2_O [12], against both Gram-negative and Gram-positive bacteria are well known. These oxides have been used to decontaminate air, water, soil, textiles and wounds. We emphasize that each of these use environments is different, and the results and antibacterial mechanisms of one cannot be compared to those of another.

The physicochemical characterizations of the metals we have studied have always included high resolution XPS. The spectra so obtained were separated into component peaks, using symmetric component line shapes [13], which revealed the binding energies of the separated peaks contributing to each spectrum. In this way, the metal and oxygen peaks of each surface oxide were identified. When compared to literature values of pure bulk oxides [14], they were found to be indistinguishable, indicating that the structure of the surface oxide is very close to that of the bulk oxide.

## 4. The Antibacterial Properties of Oxometallates

Oxometallates are compounds in which the metal oxide exists as the anion (e.g., cuprates, ferrates, etc.). We have not investigated oxometallates. However, some have been characterized by XPS, and are found in the NIST XPS Database [14], along with their formal oxides. Comparisons of peak positions of the oxides and oxometallates of specific metals find them at equivalent binding energies, implying similar structures.

The antibacterial properties of oxometallates have not been widely evaluated. Nonetheless, where evaluated (e.g., vanadates [15]), they show antibacterial behavior. Although such behavior may be due, in part, to the associated cations, minimum inhibitory concentration (MIC) test values [15] are similar for oxide and oxometallate, suggesting similar mechanisms.

A recent article [16] proposed the use of oxometallate (and metal oxide) nanoparticles, incorporated into membranes, for antibacterial water remediation. While the situation differs from the one presently considered (no protein corona is formed, no obvious water components capable of reducing any ions emitted from the nanoparticles, etc.), they support their proposal with references to the use of both oxometallates and metal oxides in toxicity studies.

## 5. The Surface Structures of Metal Oxides

All metal oxides [17,18] are characterized by the M–O–M structure (other structures, such as M=O and M–OH may also be present). On exposure of the oxide surface to water (or humidity), the M–O–M structure at the surface is hydrolyzed to give M–OH. The isoelectric points of metal oxides appear to depend on their electronegativities [19], and range from pH 1 to pH 12. Thus, at the pH of human blood and plasma, ~7.35, the nanoparticle surface may, depending on the metal oxide, exist mostly as M-OH_2_^+^ or M-O^-^. Both species are capable of reacting with proteins and similar structures [20,21,22].

## 6. The Protein Corona

Authors often include graphical illustrations of proteins in the act of forming coronas; they invariably show the proteins adsorbing onto the nanoparticle surface without changing form [23]. However, actual TEM photomicrographs reveal something quite different: with rare exceptions, the proteins in the corona have lost their structure and, as a result, the corona is amorphous, and totally surrounds the nanoparticle [24]. This is to be expected: The sheets and helices of proteins are formed by both bonded and nonbonded interactions. Perturbing such interactions, by adsorption onto a nanoparticle, results in changes to the unbonded structure and, in extreme cases, in complete unfolding.

Although papers identifying nanoparticle corona proteins that were adsorbed from protein-containing liquids, such as blood and plasma, are rather scarce, several [25,26,27,28,29,30,31,32,33,34] do exist. A number of points that are important to the present discussion can be gleaned from them:

- The hard corona is generally irreversibly adsorbed.
- The proteins adsorbed depend on the nanoparticle material (Ag, Au, Fe_3_O_4_, etc.).
- For a given nanoparticle material, the proteins depend on nanoparticle shape.

The first and third of these are understandable from the point of view of chemical bonding of the proteins to the functional groups available on the nanoparticle surface, and both occurrences change the bio-identity of the nanoparticle. Further, the loss of initial protein structure on adsorption means that new epitopes are formed on the corona-covered nanoparticle surface; these have the capacity to react with the bacterial surface. The second point clearly shows that the chemical character of the corona depends on the material on which it is formed.

This raises several questions on the role of the nanoparticle in the body. What is the function of the nanoparticle in the attack on the bacterium? Does it participate in the attack (e.g., release ions) or is it merely the carrier for the new corona protein epitope, which carries out the attack?

The protein corona must act, at the very least, as a partial diffusion barrier to the release of metal ions. Both blood and plasma contain components capable of reducing metal ions to zerovalent metal atoms [35]. Is it, then, possible for the nanoparticles to release ions or is its function rather to react with specific proteins from among the thousands [36] that are present in human biofluids?

The corona-forming interactions that apply to the nanoparticle must also apply to the bacterium. However, the type of corona forming around the bacterium, whether hard or soft, is presently unknown. As to whether a corona actually forms, TEM photomicrographs [37] show a shell of some sort, surrounding bacteria in blood.

Further, the coronas forming around Gram-positive bacteria are, without a doubt, different than those forming around Gram-negative bacteria. This is due to their different surface structures [38,39]:

- Gram-positive bacteria have a surface layer comprised of a thick transpeptidation-crosslinked [40] latticework of peptidoglycan.
- Gram-negative bacteria have a surface layer comprised of a lipid bilayer, whose outer leaf is composed of glycolipids, and whose inner leaf is composed of phospholipids.

In order to exert an antibacterial effect, the epitope formed by the protein corona must be able to attack the bacterial surface; this may be one reason why nanoparticles display different antibacterial behaviors against Gram-positive and Gram-negative bacteria.

## 7. The Exceptional Antibacterial Efficacy of Nanoparticle Alloys

In a recent review of Ag and Cu nanoparticles, and their mixtures and alloys [4], the conclusion was reached that the order of antibacterial efficacy was Ag ≈ Cu < a mixture of Ag + Cu < AgCu alloy. This has been confirmed in a recent investigation [41]. The authors of that study used an aqueous mixture of soluble Ag and Cu salts, which were then reduced by aqueous NaBH_4_ in the presence of polyvinyl pyrrolidone and polyvinyl alcohol. While the increased efficacy of a mixture of Ag + Cu over either Ag or Cu (a mixture of Ag + Cu > Ag ≈ Cu) may be accounted for by synergy, the increased efficacy of AgCu alloy over a mixture of Ag + Cu (AgCu alloy > a mixture of Ag + Cu) cannot.

This increased efficacy is surprising, especially since Ag and Cu are mutually immiscible [42]. Although a trace of one metal may exist in the other, introduction by intentional doping showed the dopants to be located in the grain boundaries [43,44]. Similar exceptional efficiencies, both antibacterial [45,46] and antiviral [45,46,47], have been found for oxometallates formed from two metal oxides.

Our perception of this exceptional efficacy, in light of our earlier results on the physicochemical characterizations of such nanoparticle surfaces and our discussion of the dependence of the protein corona content on the nanoparticle material (above), lead us to the following view. The chemical groups present on the surface of a nanoparticle depend on the identity of the nanoparticle, as do any residual surface groups, which depend on how it was made [48,49,50]. The chemical properties of these surface groups, in an alloy, result in reaction with a combination of specific proteins not found on the individual nanoparticles, and the formation of a corona that has an antibacterial efficacy that differs from those of the protein coronas that are formed about the individual nanoparticles of the same metals (which persist in their mixture). This suggestion, if confirmed, opens several possibilities.

First, the alloy nanoparticle does not function simply as a carrier, but as a bimetallic mediator, although not in the sense of a recent review [51], in which the bimetallic structure reorganizes so as to mediate reaction. In the present case, structural reorganization of the nanoparticle does not occur. Rather, the combination of metals results in the close proximity of two surface oxides on the nanoparticle surface, mediating the formation of a new corona, made up of a combination of proteins not found in the coronas of the individual monometallic nanoparticles.

In the case of the Ag, Cu system, the isoelectric point of CuO is ~9 [19], and that of Ag_2_O, ~12 [52]. (The reader should note that isoelectric points for a given oxide, reported in the literature, generally cover a wide range of pH values, reflecting the surface components present; as mentioned earlier, these depend on the particular fabrication process used [48,49,50]. While we believe that the values that we quote represent the oxides, we have no way of confirming this.) The values are noticeably different (pH is a logarithmic scale), both being positively charged at the pH of human blood. Each will react with different proteins, as may be verified by a comparison of the proteins in the hard coronas of the alloy and the individual metals. Thus, the combination of these two different sets of proteins results in a corona different from those of the individual metal nanoparticles. We believe that the epitope of this new corona conveys the enhanced antibacterial properties found.

Second, this view, if confirmed, opens the possibility of engineering alloy nanoparticles with even greater antibacterial efficacy, specific to a given bacterium. This can be done by changing alloy components or component ratios, as well as by surface functionalization: as noted earlier, both M-OH_2_^+^ and M-O^−^ are reactive [20,21,22], and can react with molecules having other functional groups (e.g., −COOH, −SH, etc.) capable of selectively reacting with designated blood proteins. The modulation of the contents of the corona by surface functionalization is an established fact [25,26,27,28,29,30,31,32,33,34,53].

Thus, we propose that the antibacterial efficacy of a metal-based nanoparticle, in the human body, is due to protein corona-bacterium interactions. These are enhanced and accelerated by the well-established behavior of nanoparticle surfaces [51]. This view is compatible with all of the observations which we have presented here. A recent review [54] discusses antibacterial peptides, offering several scenarios of how they may attack the bacterial surface. This further supports the view presented here.

## 8. Summary and Commercialization

We propose a new view of the antibacterial efficacy of nanoparticles. We use our earlier observations to provide new insights, and to propose that the chemical groups on the nanoparticle surface serve as a mediator for the formation of the protein corona, which attacks the bacterium. Further, the contents of the corona depend on which specific chemical groups are situated on the nanoparticle surface, which implies that alloy nanoparticles may be engineered to have even greater antibacterial efficacy, specific to each bacterium.

Insofar as we have been able to determine by our physicochemical characterizations of nanoparticle surfaces, the surface oxide is, by far, the major inorganic component. Our research on the subject indicates that, while there are batch-to-batch variations in the concentration of surface oxide, even when maintaining strict protocols [55], efficacy tests similar to those used by drug manufacturers will determine batch acceptability.

## Data Availability

Not applicable.
